# Gender, Acculturation, and Health-Related Quality of Life Among the Arab Ethnic Minority: A Syndemics Approach

**DOI:** 10.1007/s40615-025-02445-2

**Published:** 2025-04-22

**Authors:** Lior Moskovich, Anat Amit Aharon

**Affiliations:** 1 Faculty of Medical and Health Sciences, School of Medicine, Tel Aviv University, Tel Aviv, Israel; 2 Department of Nursing Science, Faculty of Medical and Health Sciences, Tel Aviv University, Ramat Aviv, P.O.B 39040, 6139001 Tel Aviv, Israel

**Keywords:** Gender disparities, Acculturation, Health-related quality of life, Arab ethnic minority, Syndemics theory

## Abstract

**Introduction:**

Gender disparities in health-related quality of life (HRQoL) among ethnic minorities remain a critical concern. HRQoL refers to the assessment of an individual’s subjective physical and psychological well-being. This study investigates these disparities within the Arab ethnic minority in Israel, focusing on the interplay between acculturation styles and behavioral syndemic factors. The syndemics approach, which considers the interaction of co-occurring health conditions within specific social and environmental contexts, serves as the theoretical foundation.

**Methods:**

A cross-sectional design was employed to collect data from 305 participants among the Arab ethnic minority using a validated self-report questionnaire. Key variables measured included acculturation style, syndemic structure based on the cumulative impact of the four health-related behaviors, and HRQoL. Statistical analyses involved a one-way MANCOVA. A hierarchical linear regression was conducted to determine the associations between the variables.

**Results:**

The findings indicated significant gender differences, with Arab women exhibiting poorer HRQoL than men, particularly regarding physical fitness, daily activities, and social activity. Marginalized and integrated acculturation styles, higher syndemic severity, and chronic illness were associated with lower HRQoL. The regression model was significant, explaining 16.8% of the variance in HRQoL.

**Conclusion:**

Addressing health conditions in isolation may be insufficient without considering the broader social and environmental influences. Implementing culturally tailored intervention programs incorporating acculturation challenges and syndemic interactions is recommended to enhance HRQoL among ethnic minorities. These findings underscore the importance of “upstream” policies to reduce health disparities and promote equity.

**Supplementary Information:**

The online version contains supplementary material available at 10.1007/s40615-025-02445-2.

## Background

Israel’s population is characterized by ethnic diversity, with the Arab population representing the largest ethnic minority group alongside the Jewish majority and the Christian and Druze minorities. Acculturation style refers to the strategies utilized by individuals or groups to adjust to the dominant culture. The acculturation style of ethnic minority citizens in general society may have a crucial impact on their health behavior and outcomes [1, 2]. Studies from Israel indicate significant social, economic, and health inequalities between the Arab ethnic minority and the majority population. Furthermore, disparities in these domains are also observed between Arab women and men [3]. Nevertheless, research focusing specifically on gender-based health disparities within Israel’s Arab minority remains limited.

To address this gap, the study used the syndemics theory, which considers the interaction of co-occurring health conditions within specific social and environmental contexts, as a framework for analyzing and interpreting the findings. The syndemics theory offers a holistic approach to addressing public health challenges from a social, political, and economic perspective. It provides a comprehensive lens for analyzing the compounded effects of acculturation style, health behavior, and social inequality on HRQoL in this population.

### The Arab Ethnic Minority in Israel

Israeli society is characterized by its plurality of diverse multicultural and ethnic groups. At the end of 2022, Israel’s population was estimated at approximately 9.662 million. The Jewish population comprised 74% and the Arab population 21.1% of this number, the latter equaling about 2.039 million people, of whom approximately 85% are Muslims [4]. In general, Israel’s Arab ethnic minority is characterized by lower socioeconomic and education levels and poorer health indicators compared to the Jewish population [5]. Employment is an essential factor that impacts socioeconomic status [6]. In the last two decades, the Arab ethnic minority, and especially Arab women, has joined the trend of increased employment, contributing to their integration into Israeli society. In 2012, only 29.3% of Arab women were employed, compared to 42.8% in 2022 [7]. Despite the positive change, the labor force participation and employment rates in the Arab community remain low compared to the general population, particularly among Arab women and young people under the age of 24. The main barriers include proficiency in Hebrew, lack of education and occupational diversity, cultural and gender barriers, lack of work experience, and others [7]. Studies worldwide found that integrating low socioeconomic populations (including minorities) into the workforce mitigates socioeconomic and health disparities [8, 9]. Another significant transformation Arab society has experienced is particularly evident in the growing participation in higher education. The rate of Arab students pursuing a bachelor’s degree in academic institutions increased from 10% (22,268) in 2010 to 18.3% (43,454) in 2020. The same trend is also true of master’s degree and PhD students [4]. A nursing academic degree is considered very popular and respectable among Arab women and men [10]. Research suggests that higher education and professional careers can empower women in traditionally patriarchal societies, such as the Arab ethnic minority in Israel [3]. Higher education has been found to be associated with lower morbidity and mortality and higher well-being [11, 12].

As equal citizens of Israel, members of the Arab ethnic minority receive the same health services as the majority population. The National Health Insurance Law 1995 determined that all citizens are covered by health insurance in one of four Health Maintenance Organizations (HMOs) [13]. HMOs operate preventive and curative health services through community clinics nationwide, providing equitable and accessible healthcare to all citizens. However, there may be reduced accessibility to these clinics in peripheral regions such as the Negev area [14]. Many studies examined the health disparities between the Arab ethnic minority and the Jewish majority [15, 16]. However, there is a paucity of studies specifically addressing gender health disparities within the Arab ethnic minority in Israel. For example, Arab men exhibit higher smoking rates compared to Arab women and the general population [14].

### Health-Related Quality of Life and Minorities

Health-Related Quality of Life (HRQoL) is a multidimensional concept that encompasses physical functioning, mental and emotional status, social functioning, pain and discomfort, well-being, and other aspects related to an individual’s health. It is used to assess the impact of health status on a person’s overall quality of life [17].

Variations in HRQoL are evident across different countries, populations, diseases, ages, and genders. Comparative studies across diverse populations and demographics may provide valuable insights into the underlying causes of HRQL disparities and inform targeted interventions to address these variations and inequities [18, 19]. When examining HRQoL among minority populations, notable impairments are evident. A study conducted among African American men in Cleveland revealed significantly lower HRQoL across six health domains: physical functioning, general health, social functioning, role-emotional, mental health, and role-physical, compared to the general US population [20]. Another study investigating gender-related differences in HRQoL among patients with colorectal cancer in Iran found that women are more adversely affected than men in terms of impaired physical and social functioning following the onset of cancer [21]. These findings may suggest pervasive health disparities and measurable differences in disease outcomes, which may elevate mortality rates within vulnerable populations [22].

### Acculturation, Health, and Minorities

Acculturation is defined in many ways. There is a consensus that acculturation is an interactive and continuous process encompassing physical, psychological, social, emotional, and economic changes [23, 24]. The changes relate to affective, behavioral, and cognitive aspects occurring among specific groups such as immigrants, refugees, and ethnic minority populations [24].

The concept of acculturation strategy was initially introduced by psychologist John W. Berry in 1997. Berry’s acculturation model delves into the methods individuals employ to assimilate into a host or dominant society [25]. This framework has been extensively researched among immigrant and diverse ethnic minority populations [26–28]. Berry’s acculturation model posits that individuals encountering a new culture navigate a complex interplay between their heritage culture and the host or dominant culture. This model is predicated on two dimensions [27]:

- Affinity with the heritage culture**:** This dimension explores the degree to which an individual maintains their original cultural identity, customs, and values.
- Affinity with the dominant culture**:** This dimension examines the extent to which an individual incorporates the dominant culture’s norms, behaviors, and values.

The intersection of these dimensions yields four distinct acculturation strategies:

- **Assimilation:** Individuals fully embrace the dominant culture while relinquishing their heritage culture.
- **Separation:** Individuals maintain their heritage culture while rejecting the dominant culture.
- **Integration (or biculturalism):** Individuals successfully balance their heritage and dominant cultures, achieving a harmonious coexistence.
- **Marginalization:** Individuals reject their heritage and dominant cultures, experiencing a sense of alienation and disconnection.

Berry (2011) emphasizes the dynamic nature of acculturation, recognizing that individuals may adopt different strategies in various life domains and over time. One can adhere to their heritage culture in their home and family (separation style) while adopting the dominant culture in the workplace (assimilation style).

Studies have found an inconsistent association between acculturation style and health behavior and health outcomes. A study among the Arab minority in Israel found a positive association between assimilation and separation acculturation style and adherence to COVID- 19 vaccination [1]. A study from the USA found that Korean immigrants with higher assimilation orientation were more likely to have healthier dietary behavior and decreased body image discrepancy compared to Korean immigrants with a separation orientation [29]. A study that compared health behavior among traditional Mexican Americans (born in Mexico) and acculturated Mexican Americans (born in the USA) found that traditional Mexican Americans with separation acculturation have significantly worse health behavior (smoking, exercise, and fruit/vegetable consumption) than Mexican Americans born in the USA who display integration acculturation. These findings were found among both genders [30]. Conversely, a study among young Latin women found that a separation acculturation style was associated with a high risk of sexual behavior, for example, multiple sex partners and less condom use [26]. Finally, no significant association was found between acculturation and knowledge and attitudes among Latina women from Florida regarding HPV vaccination [31]. It is worth noting that acculturation was measured using different methods, which may impact the results of these studies.

### The Syndemics Framework

The term “syndemics” derives from a combination of two Greek-rooted words: “synergy,” meaning working together, and “epidemic,” referring to widespread disease. Therefore, syndemics describes the interaction of multiple epidemics or health issues that amplify their combined effects on a population. Merrill Singer’s (health anthropologist) syndemics theory posits that diseases and health conditions are often interconnected and exacerbated by social and environmental factors. Instead of looking at each disease independently, this theory examines how multiple diseases or health conditions cluster together within a vulnerable population or community, interacting in harmful ways regarding this given population [32]. The basis of the syndemics theory consists of interactions between three components: the co-occurrence of two or more diseases or health conditions in a given vulnerable population, the physical or psychological interrelationship between the diseases or health conditions, and the social-cultural context that impacts the population in their residential environment. This process may increase the disease burden or harmful health conditions in the population or community [33, 34]. Namely, the process of syndemics consequences worsens health outcomes among vulnerable populations or communities. By understanding the complex interplay between diseases or health conditions and social conditions, syndemics theory offers a holistic approach to addressing public health challenges from a social, political, and economic perspective. These factors are essential for promoting population health [35]. The social context that impacts health conditions, inequality, and diseases can comprise poverty, stress, ethnic inequality, immigration, structural violence, stigmatization, environmental pollution, structural discrimination, and others. These factors are associated with disease clustering and physical and mental health [33, 36] Therefore, the syndemics process may explain health disparities between people or communities [37].

The current study focused on the Arab ethnic minority in Israel as a vulnerable population, reflected by their lower socioeconomic status compared to the general population as described previously, with a gender comparison within the given population. Two co-occurring social-health conditions were examined. The first was a syndemic construct developed from four independent components: health behavior regarding smoking, exercising, receiving the seasonal flu vaccine, and healthy nutrition. The second was the acculturation style according to Berry and Hou’s (2016) definition. It was hypothesized that an adverse interaction between the syndemics construct and acculturation style would be associated with impaired health-related quality of life among the given vulnerable population.

In light of the syndemics theory, the aims of the study were twofold: (a) to explore gender differences in acculturation style, syndemics construct, and health-related quality of life among the Arab ethnic minority in Israel; (b) to explore the association between syndemic construct, acculturation style, and health-related quality of life among the Arab ethnic minority in Israel.

## Methods

### Study Design

This is a cross-sectional study with a quota sample.

### Data Collection and Sample Size

The study recruited participants through an online polling service, *I-Panel* (https://www.ipanel.co.il/en/). This service is known to be reliable and representative of the Israeli population [38]. The polling service is a member of the European Society for Opinion and Marketing Research (ESOMAR). Adhering to international data collection standards ensures the highest data quality [39]. One such measure involves including dummy questions to verify respondent attention and data accuracy. A quota sample procedure was utilized to select the research sample. The strata were defined based on Israel’s geographical regions, with the allocation of sample units to each stratum proportional to the relative size of the Arab population within that region. The questionnaire was sent randomly to eligible Israeli Arab adults who could read Hebrew or Arabic by email or SMS. Participants received a small monetary compensation for their participation.

The sample size was calculated using G*Power version 3.1.9.7 [40]. One-way MANCOVA parameters were included: effect size *f* = 0.15 (medium), power = 0.80, *α* = 0.05, number of groups = 2, number of covariances = 4. The minimum sample size calculated was 269 participants. The final sample size consisted of 305 participants. Post hoc power analysis revealed a power of 0.86 with this sample size.

The data collection was conducted during July 2023. The reporting adhered to STROBE guidelines.

### Measurements

The study used a closed self-report questionnaire with four sections, as follows:

*Personal Details*: Gender, age, marital status, educational level, income, and religiosity.

*Acculturation Style*: The acculturation style was measured using two questions. First, the participants were asked to score their affinity with the Israeli major culture and, second, their affinity with their Arab heritage. The questions were adapted from [41] acculturation questionnaire. Responses were from 1 = “extremely strong affinity” and 5 = “extremely weak affinity.” As Dona and Berry (1994) suggested, responses 1 + 2 were aggregated to a “yes” answer (= 1). Responses 3 + 4 + 5 were aggregated to a “no” answer (= 0). Through the two questions and the two aggregations of the responses (yes/no), four acculturation styles were defined, as follows: (A) If the answer to both questions was “yes,” the score was “2,” meaning integration acculturation style. (B) If the answer to the first question was “yes” and to the second question “no,” the score was “1,” meaning assimilation acculturation style. (C) If the answer to the first question was “no” and to the second question “yes,” the score was “1,” meaning separation acculturation style. (D) If the answer to both questions was “no,” the score was “0,” meaning marginalization acculturation style (supplemental material 1). Notably, in the current study, the correlation between affinity with the Israeli major culture and affinity with Arab heritage was significant but low (*r* = 0.18, *p* < 0.001). This suggests that the two dimensions are independent and that high affinity with Israeli culture does not replace affinity with the Arab heritage culture, and vice versa. There is a continuum between the two cultural affinities [27].

*Health-Related Quality of Life (HRQOL)*: Health-related quality of life was measured using the Functional Health Assessment Charts (‘COOP/WONCA charts”) [42]. This questionnaire assesses one’s subjective health functional capacity during a given period. Functional health status “is an aspect of health that, in turn, is an aspect of quality of life” [42]. The Functional Health Assessment Charts were used in adequate studies across counties and multiple patients [43, 44]. The questionnaire assesses six domains of functional status: physical fitness, daily activities, mental function, social activities, general health, and change in health status. The questionnaire was translated and validated in more than 20 languages, including Hebrew and Arabic. The reliability of the questionnaire was assessed by test–retest with satisfactory reliability [42]. In the current study, internal consistency was 0.72.

The participants were asked to score their functioning during the last 2 weeks. Answers were on an interval scale of 1 to 5. Each item had its own answers. For example, “What was the hardest physical activity you could do for at least 2 min?” The answers were from 1 = very heavy, for example, run, at a fast pace, through 5 = very light, for example, walk, at a slow pace or not able to walk. Mean and SD were calculated for each item and for the entire questionnaire. Higher scores mean worse health functioning. Functional health status was the health outcome.

*Syndemic Construct*: The syndemic construct was calculated based on four independent health behavior subscales. The health behavior questionnaire was adapted from the Knowledge, Attitude, and Practice (KAP) survey conducted in Israel once every few years [45]. The current study used seven questions from the KAP survey: one item regarding smoking cigarettes, one item regarding exercise, one item regarding flu vaccination, and four items regarding healthy nutrition (nutrition subscale). The nutrition questionnaire consisted of food consumption habits encompassing family eating habits, drinking sugar juices, eating whole bread, and eating industrialized food. All seven questions had four possible responses, where a higher rating indicated worse health behavior. For example, responses to the question “How often do you exercise?” ranged from 1 = “at least 150 min per week” to 4 = “no exercise at all.” The health behavior questionnaire was utilized to calculate the syndemic structure, as will be explained below.

### Statistical Analysis

Descriptive statistics was used to describe the sample, divided into men and women. For categorical variables, a chi-square test was performed, and for continuous variables, a *t*-test was performed. Normal distribution was examined with skewness and kurtosis parameters, suggesting a normal distribution. A one-way MANCOVA was performed to analyze gender differences regarding functional health status for each of the six domains and for the entire questionnaire, controlling for age, education, marital status, income, health status, and religiosity. For each domain, the effect size was calculated (*η*^2^). Basic assumptions for the MANCOVA were tested with no violation [46].

The syndemic construct was calculated and driven by four independent health behavior variables. Smoking, exercising, and receiving the flu vaccine in the last year were recoded as dichotomized variables with 0/1 scores. For example, receiving the flu vaccine had four possible responses. The responses “received the vaccine” and “intention to receive the vaccine” were aggregated to a score of “1.” The responses “did not receive the vaccine” and “I have no intention to vaccinate” were aggregated to a score of “0.” The four nutrition items in the nutrition questionnaire were recoded as aforementioned and then summarized into one variable, “nutrition habits,” with a response range of 0–4. In the next step, the nutrition habits variable was recoded as a dichotomized variable, where the 0–1 response was defined as “0” (meaning zero to one nutrition risk) and responses 2–4 were defined as “1” (meaning two or more nutrition risk factors). At the end of this process, the syndemic construct consisted of four variables with a score of 0/1. Two dimensions of the syndemic construct were calculated: the syndemic score and the syndemic severity score, as suggested by Martinez et al. (2017).

#### Syndemic Score

The syndemic construct was recoded as three categories: scores of 0–1, meaning that the participant has zero to one health risk (low-risk health behavior); score 2, meaning that the participant has two health risk factors (moderate-risk health behavior); scores 3–4, meaning that the participant has three to four health risk factors (high-risk health behavior). A chi-square test examined associations between gender, socioeconomic and acculturation style variables, and the three syndemic score categories.

#### Syndemic Severity Score

The syndemic construct is based on four health behaviors: smoking, exercising, receiving the flu vaccine in the last year, and nutrition habits. All were rated on a scale of 1–4. The values for each participant were summed for a response range of 4–16; the higher the scores, the higher the severity of the risk factors. A *t*-test was performed to analyze differences in syndemics severity by gender and socioeconomic variables. An ANOVA was performed to analyze differences in acculturation style by syndemics severity. Cohen’s *d* was calculated as effect size.

Finally, a three-step hierarchical linear regression was conducted to examine the association between the research variables (gender and socioeconomic variables, four acculturation styles, syndemic scores, and syndemic severity) and functional health status. Using hierarchical linear regression makes it possible to identify the unique contribution of each of the variables. Before performing the regressions, the acculturation styles were recoded as a dummy variable. Each acculturation style was compared to the others, for example, marginalization = 1 and other styles = 0. It was decided that the assimilation style would not be included in the equation, as only 14 participants presented this acculturation style.

In the first step, the socioeconomic variables were entered. Acculturation styles were inserted in the second step, and the syndemic score and severity were inserted in the third step. Parameters of VIF < 10, tolerance > 0.2, Durbin-Watson statistics 1.94, and normal probability plots suggested no multicollinearity and normal distribution of the residuals [46]. The significance of the models and the explained variance (*R*^2^) were calculated. SPSS package version 29 was used for the analysis.

### Ethical Consideration

The university Ethics Committee approved the study (#0002629–1). The questionnaire began with an explanation of the research topic and an invitation to participate. Participants had to note their consent to participate in the study. Participants were assured of their anonymity and complete confidentiality and were informed of their right to withdraw from the study at any point.

## Results

Altogether, there were 305 participants in the study, 56.4% of whom were women. No significant gender differences were found in the socioeconomic variables, except that women were more religious than men (79.7% vs. 60.9%, *p* < 0.001). Only a few participants displayed the assimilation acculturation style (4.6%), but men displayed this style statistically significantly more than women (7.5% vs. 2.3%, *p* = 0.03) (Table 1).

**Table 1:** Sample characteristics (*n* = 305)

Variable	All sample (*n* = 305)	Men (*n* = 133, 13.6%)	Women (*n* = 172, 56.4%)	*χ*^2^	*p* =
Marriage status				1.06	0.30
Marriage	171 (56.1)	79 (59.4)	92 (53.5)		
Not marriage	134 (43.9)	54 (40.6)	80 (46.5)		
Income				1.78	0.18
Below average-average	238 (78.0)	99 (74.4)	139 (80.8)		
Above average	67 (22.0)	34 (25.6)	33 (19.2)		
Education				2.77	0.09
Not academic	126 (41.3)	62 (46.6)	64 (37.2)		
Academic	179 (58.7)	71 (53.4)	108 (62.8)		
Religiosity				12.93	< 0.0001
Secular	87 (28.5)	52 (39.1)	35 (20.3)		
Religious-very religious	218 (71.5)	81 (60.9)	137 (79.7)		
Chronic disease				0.63	0.42
No	271 (88.9)	116 (87.2)	155 (90.1)		
Yes	34 (11.1)	17 (12.8)	17 (9.9)		
Acculturation style					
Marginalization	67 (22.0)	30 (22.6)	37 (21.5)	0.04	0.82
Separation	140 (45.9)	53 (39.8)	87 (50.6)	3.48	0.06
Assimilation	14 (4.6)	10 (7.5)	4 (2.3)	4.61	0.03
Integration	84 (27.5)	40 (30.1)	44 (25.6)	0.76	0.38
	*M* (SD)	*M* (SD)	*M* (SD)	*t*	*p* =
Age	32.6 (10.82)	32.5 (10.48)	32.6 (11.11)	− 0.17	0.86

A one-way MANCOVA was conducted to determine the effect of gender on the six variables related to the functional health status questionnaire, controlling for age, education, income, marital status, and religiosity. Box’s M test indicated homogeneity of variances and covariances (*p* > 0.001), and Wilke’s *Λ* (Lambda) was insignificant (Wilke’s *Λ* = 0.96, *F* = 1.55, *p* = 0.16), suggesting no violation of the assumption of homogeneity of covariance matrices. The MANCOVA showed a statistically significant gender difference in physical fitness (mean = 2.44, SD = 1.28, vs. mean = 2.74, SD = 1.25, *p* = 0.04, *η*^2^ = 0.016), daily activities (mean = 2.11, SD = 1.17, vs. mean = 2.49, SD = 1.20, *p* = 0.03, *η*^2^ = 0.016), and social activities (mean = 2.10, SD = 1.16, vs. mean = 2.48, SD = 1.29, *p* = 0.02 *η*^2^ = 0.016), as well as in functional health status as a whole (mean = 2.32, SD = 0.71, vs. mean = 2.55, SD = 0.74, *p* = 0.03, *η*^2^ = 0.016). Arab women show impaired physical fitness and daily and social activities compared to Arab men, with an effect size of small to medium (Table 2).

**Table 2:** Gender differences in functional health status, MANCOVA^±^

Functional health status variable	Man, *M* (SD)	Women, *M* (SD)	*F*	*η*^2^
Physical fitness: What was the hardest physical activity you could do for at least 2 min?	2.44 (1.28)	2.74 (1.25)	3.25*	0.011
Mental function: How much have you been bothered by emotional problems such as feeling anxious, depressed, irritable, downhearted, and sad?	2.35 (1.20)	2.52 (1.33)	0.84	0.003
Daily activities: How much difficulty have you had doing your usual activities or tasks, both inside and outside the house because of your physical and emotional health?	2.11 (1.17)	2.49 (1.20)	4.90*	0.016
Social activities: Has your physical or emotional health limited your social activities with family, friends, neighbors, or groups?	2.10 (1.16)	2.48 (1.29)	5.12*	0.017
Change in health status: How would you rate your overall health now compared to 2 weeks ago?	2.73 (0.81)	2.80 (1.01)	0.001	0.00
General health: How would you rate your health in general?	2.19 (0.97)	2.29 (1.02)	1.30	0.004
The whole questionnaire: COOP questionnaire	2.32 (0.71)	2.55 (0.74)	5.13*	0.017

^±^Controlling: age, marital status, education, income, religiosity, chronic disease

^*^p < 0.05 (two-tailed)

Men had statistically significantly higher syndemic scores than women: 66.9% had a syndemic score of 3–4, while only 54.1% of the women had such a score (***χ***^2^ = 8.41, *p* = 0.01). No other associations were found between socioeconomic variables, acculturation style, and syndemic scores (supplemental material 2).

A *t*-test and ANOVA assessed differences in syndemic severity by gender and SE variable. Women had statistically significantly lower syndemic severity than men, with a large effect size (mean = 5.65, SD = 2.56 vs. mean = 6.28, SD = 2.81, *p* = 0.04, *η*^2^ = 2.67). No other differences in SE variables were found (supplemental material 3).

A three-step hierarchical linear regression was conducted to assess the factors that explain functional health status. The third step revealed that being a woman, non-married, very religious, and with a background chronic disease were associated with higher scores for functional health status, meaning lower health-related quality of life (*β* = 0.13, 95% CI = 0.03–0.36; *β* = 0.21, 95% CI = 0.14–0.50;* β* = 0.13, 95% CI = 0.04–0.40; *β* = 0.15, 95% CI = 0.09–0.62, respectively). Moreover, marginalization and integration acculturation and syndemic severity were statistically significantly associated with higher scores of functional health status, which means lower health-related quality of life (*β* = 0.26, 95% CI = 0.05–0.87; *β* = 0.25, 95% CI = 0.17–0.83, *β* = 0.27, 95% CI = 0.03–0.12, respectively). Syndemic severity and marginalization acculturation had the highest impact on functional health status. The model was significant (*p* < 0.001) and explained 16.8% of the variance (Table 3).

**Table 3:** Hierarchical linear regression explaining functional health status (3rd step)

Variable*	*β* (95% CI)	*p* =
Gender	0.13 (0.03–0.36)	0.02
Age	0.11 (− 0.001 to 0.01)	0.07
Marital status	0.21 (0.14–0.50)	< 0.001
Education	0.02 (− 14 to 0.19)	0.32
Religiosity	0.13 (0.04–0.40)	0.01
Income	− 0.06 (− 0.63 to 0.14)	0.21
Chronic disease	0.15 (0.09–0.62)	0.009
Acculturation		
Separation	0.19 (− 0.09 to 0.69)	0.14
Marginalization	0.26 (0.05–0.87)	0.02
Integration	0.25 (0.17–0.83)	0.04
Syndemic score	0.08 (− 0.28 to 0.10)	0.36
Syndemic severity	0.27 (0.03–0.12)	0.003
Sig. *F*	< 0.001	
*R*^2^ (%)	16.8	

^*^Gender: men = 0, women = 1; marital status: married = 0, not married = 1; education: not academic = 0, academic = 1; religiosity: secular = 0, very religious = 1; income: below average = 0, above average = 1; chronic disease: no = 0, yes = 1; acculturation: separation = 1, all others = 0; marginalization = 1, all others = 0; integration = 1, all others = 0

## Discussion

The current study examined gender health differences in HRQoL within the framework of the syndemic theory among members of the Arab ethnic minority living in Israel. The findings indicate that Arab women have lower syndemic scores (meaning a lower health risk) and lower syndemic severity (meaning a lower severity of health risk factors) compared to Arab men. Nevertheless, Arab women demonstrated a lower functional health status compared to Arab men, particularly in physical fitness, daily activities, and social activities. Moreover, gender emerged as a significant explanatory factor for functional health status, with women consistently associated with poorer functional health status, meaning lower HRQoL.

There is a consensus that increased health risk factors are associated with higher morbidity and mortality [47]. Worldwide, studies have demonstrated that gender differences in health lifestyles impact health outcomes. Studies have argued that the differences between men and women are not merely attributable to gender-biological variations but are rooted in gender-related social factors [48]. For example, in the context of chronic illness, social and family support is critical for coping with the illness. Research suggests that men tend to receive more support from their family than do women, which often results in better responses to illness (for example, faster recovery with fewer complications) [49]. Overall, women are frequently positioned as primary caregivers within the family, which imposes physical and emotional demands and may negatively impact their health and well-being [50]. This caregiving burden is ultimately reflected in HRQoL deterioration [51]. Additionally, there is growing evidence that women with traditional risk factors such as obesity, diabetes, hypertension, and smoking have a relatively higher risk of developing cardiovascular disease than men with similar profiles [52]. These broader findings are consistent with our study and reinforce the idea that lower HRQoL among women is not only a reflection of behavioral risk burden but is influenced by structural, cultural, and psychosocial stressors.

Cherepanov et al. argue that gender disparities in HRQoL can be attributed to sociodemographic and socioeconomic factors such as income, marital status, and education. Their study found that women, particularly those with lower income, lower educational attainment, and unmarried status, reported lower HRQoL than men when controlling for age and race [53]. Similarly, another study examined racial disparities in HRQoL found that Black women reported lower HRQoL compared to White women. In contrast, Black men did not differ significantly from White men [54]. These findings suggest that minority women may experience worse HRQoL, resulting from the intersection of gender and ethnicity. Another important factor contributing to lower HRQoL is the discrimination experienced by ethnic minorities, which has been shown to have particularly negative effects on women’s mental health [55, 56]. These findings align with the current study in which Arab women were found to engage in less daily physical and social activities compared to Arab men, reflecting lower overall HRQoL among women.

The current study found that syndemic severity was associated with lower functional health status. In contrast, the syndemic score was not significantly associated with functional health status. Studies have shown that multi-risk factors are associated with impaired health outcomes and higher disease burden across countries [56]. For example, Martinez et al. [26] examined the syndemic severity of substance abuse, intimate partner violence, and depression symptoms among young Latina women and the association with healthy sexual behavior. Their study found that women with higher syndemic severity had a 1.68-fold increased risk of having multiple sex partners compared to women with lower syndemic severity. Another study among multi-gender populations found a 4.32-fold increased risk of harmful alcohol use and a 3.09-fold increased risk of high-risk cocaine use associated with poor adherence to HIV pre-exposure prophylaxis compared to participants with lower levels of risk behavior [57]. From a syndemic perspective, the severity of risk factors or symptoms with mutually bidirectional biological or psychosocial interactions may lead to a compounded burden on the health of a specific population [35]. These findings from the literature align with the present study, emphasizing that the severity of health risk factors, rather than simple risk accumulation, plays a critical role in shaping health outcomes.

In addition to syndemic severity, the current study found that marginalization and integration acculturation styles were associated with lower functional health status, indicating reduced HRQoL. Marginalization refers to a state where individuals feel excluded or alienated from their heritage and dominant cultures. They may experience a lack of belonging in both contexts. Integration acculturation style involves maintaining one’s heritage culture while actively participating in the dominant culture. Individuals who adopt this style often seek to balance their cultural identities [25]. While integration can constitute a positive cultural competence [58] and has positive effects on mental health [59], in certain contexts, both marginalization and integration style may lead to experiences of discrimination, prejudice, social isolation, economic shortage, and increased psychosocial and acculturative chronic stress among minority populations [58, 60]. These persistent social stressors may translate into deficient health behaviors such as poor dietary habits, heavy smoking, and physical inactivity. Over time, these health behaviors increase the risk for cardiovascular diseases, obesity, cancer, and even cognitive decline in aging [61]. These findings suggest that even adaptive acculturation strategies may have negative health consequences when situated within broader inequitable sociodemographic environments.

The association between psychosocial and acculturative chronic stress is particularly impactful among ethnic minorities, immigrants, and vulnerable populations [62]. This mechanism of impaired health outcomes deepens existing health disparities between ethnic minority populations and the dominant population [61]. However, not all aspects of acculturation exert a negative influence. A study by Gonzalez-Guarda et al. [63] among Latinx immigrants in the USA found that ethnic identification with one’s heritage culture is a protective factor against negative health behavior (heavy drinking, drug use, intimate partner violence) and health outcomes (depression and anxiety). Conversely, affinity with the dominant American culture (called Americanism, similar to integration and assimilation style) was associated with increased acculturative stress and a higher risk of negative health behaviors and outcomes.

In a systematic review of 21 articles, [59] reported that no single acculturation style was found to be significantly associated with symptoms of depression among migrant populations. While most of the studies in the systematic review found an association between marginalization style and anxiety and depression symptoms, several studies found that the other acculturation styles (including integration) were also found to be related to symptoms of depression (Choy et al., 2021). Notably, some studies found no association between acculturation and health outcomes. For example, among a sample of Latinx adults born in the USA, no association was found between acculturation and self-reported physical health [64].

A further plausible explanation for the association between integration acculturation and lower functional health status might be found in the analysis of [65], who studied acculturation processes among minorities in Israel. They suggested that biculturalism among Arabs is characterized by low integration into the Israeli identity and that the Arab and Israeli identities are negatively correlated. The Arab identity is the more relevant and salient in the individual’s daily life within their community. The Israeli identity may be in the front only when interacting with the dominant culture. The researchers concluded that integration acculturation among the Arab ethnic minority in Israel does not promote subjective well-being. Moreover, [66] found that separation acculturation among the Arab ethnic minority in Israel was associated with high well-being but not the integration style. It seems that this constant negotiation between the two identities can create a sense of “in-betweenness,” leading to increased acculturative stress and, ultimately, a decline in health-related quality of life.

Finally, the current study found a positive association between religiosity, married status, and better functional health status. These findings corroborate previous studies that found that spiritual beliefs and family support are protective factors for mental and physical health among ethnic minorities [58, 67, 68]. The Arab ethnic minority has a higher rate of religious people, and their marriage rate is higher compared to the Jewish majority in Israel [69]. These findings suggest, alongside the acculturation and syndemic factors, that higher religiosity and family support in the cultural context are important for health-related quality of life.

## Limitations

The current study has several limitations. Participants were recruited through an internet polling service. While quota sampling was used to enhance the sample’s representativeness of the Israeli Arab population [38], the overrepresentation of individuals with academic degrees (approximately 59%) compared to their proportion in Israel’s overall Arab ethnic minority may limit the generalizability of the findings. Despite previous research suggesting minimal differences across recruitment methods [70], this discrepancy warrants further investigation of potential selection biases. Also, the current study used Berry’s acculturation model [27], which is one way of measuring acculturation. The multiple ways of measuring acculturation make it difficult to compare the research findings and populations studied. In addition, worldwide studies were conducted mostly among immigrants and less among ethnic minorities; however, some were conducted among immigrants born in the host country with local citizenship (e.g., Gonzalez-Guarda et al., 2023). The current study focused on members of the Arab ethnic minority who have Israeli citizenship. The comparison with immigrants, some of whom do not have local citizenship, may impact the generalizability of the findings.

Overall, these findings highlight the importance of culturally sensitive health interventions and policies aimed at improving the quality of life among minority populations, particularly Arab women in Israel. The relationship between syndemic severity, acculturation stress, and reduced HRQoL underscores the need for multilevel approaches addressing individual behaviors and structural conditions. Public health strategies should adopt a syndemic framework to tackle interconnected stressors like discrimination, economic hardship, and cultural dissonance. Interventions fostering supportive community environments, enhancing social support, and reducing cultural and systemic barriers to care are essential. Health services must reflect Arab women’s cultural values and experiences, integrating mental health, social support, and chronic disease prevention. At the same time, greater attention should be given to the role of identity negotiation and acculturation dynamics in shaping health behavior and outcomes, ensuring that integration does not become a source of internal conflict or stress.

## Conclusion

Health behavior and acculturation style are syndemic phenomena examined among Arab ethnic minorities living in Israel. Syndemic severity, marginalization, and separation acculturation styles are associated with lower health-related quality of life among the Israeli Arab ethnic minority. The construct of syndemic severity is a meaningful factor rather than merely the number of risk factors (the syndemic score). Among the Arab ethnic minority, women are at high risk for lower HRQOL compared to men. The current research findings illustrate the complex and multifaceted relationship between acculturation among ethnic groups, acculturation coping styles, and health outcomes. This reinforces the need to mitigate health disparities between ethnic groups, the dominant population, and between men and women within a given population. Moreover, the health system and health policy should treat men and women differently and embrace gender-related and culture-related intervention and prevention programs.

Syndemic theory suggests that addressing illnesses or health conditions in isolation may be less effective unless the social and environmental factors contributing to harmful interactions are addressed as well. Fundamental factors that create health gaps must be addressed, whether this involves differences between genders or population groups. “Upstream” programs and social-health-economic policies that aim to reduce discrimination, social isolation, etc. can contribute to mitigating health disparities. Policymakers and healthcare providers should consider the impact of acculturation, with all its nuances, on health disparities. Hence, implementing a culturally adapted intervention program to meet personal needs and cultural coping methods is important. These insights will positively affect health-related quality of life among ethnic minorities.

## Supplementary Information

Below is the link to the electronic supplementary material.

Supplementary file1 (DOCX 63 KB)

## Data Availability

The data that support the findings of this study are not openly available due to reasons of sensitivity and are available from the corresponding author upon reasonable request. Data are located in controlled access data storage at the University.
