# Supplementary material for: Gender, Acculturation, and Health-Related Quality of Life Among the Arab Ethnic Minority: A Syndemics Approach
Source: J Racial Ethn Health Disparities. 2025 Apr 22;13(4):2641–52. doi: 10.1007/s40615-025-02445-2 (PMC13346293; doi:10.1007/s40615-025-02445-2)
Supplement: Supplementary file 1 — Supplementary file1 (DOCX 63 KB) [file 40615_2025_2445_MOESM1_ESM.docx]

**Supplemental materials 1-3**

**Supplemental material 1:** Scoring of acculturation style

|  | | | 1 Extremely strong | 2  Strong | | 3  Slightly strong | 4  Slightly weak | 5  Extremely weak | |
| --- | --- | --- | --- | --- | --- | --- | --- | --- | --- |
|  |  |  | Yes=1 | | | No=0 | | | |
| To what extent do you identify with the Israeli culture? | Yes=1 | Integration  (score=2) | | | Assimilation  (score=1) | | | |  |
| To what extent do you identify with the Arab culture? | No=0 | Separation  (score=1) | | | Marginalization  (score=0) | | | |  |

Source:

Dona G. & Berry J.W. (1994). Acculturation Attitudes and Acculturative Stress of Central American Refugees. *International Journal of Psychology, 29*(1), 57-70.

**Supplemental material 2**: Sample characteristics by syndemic score

| Variable | Syndemic score 0-1 | Syndemic score 2 | Syndemic score 3-4 | **χ2** | *p=* |
| --- | --- | --- | --- | --- | --- |
|  | N (%) | N (%) | N (%) |  |  |
| Gender  Men  Women | 15 (11.3)  15 (8.7) | 29 (21.8)  64 (37.2) | 89 (66.9)  93 (54.1) | 8.41 | 0.01 |
| Marital status  Married  Not married | 17 (9.9)  13 (9.7) | 58 (33.9)  35 (26.1) | 96 (56.1)  86 (64.2) | 2.31 | 0.31 |
| Income  Below average/average  Above average | 27 (9.3)  3 (21.4) | 91 (31.3)  2 (14.3) | 173 (59.5)  9 (64.3) | 3.32 | 0.19 |
| Education  Not academic  Academic | 16 (12.7)  14 (7.8) | 32 (25.4)  61 (34.1) | 78 (61.9)  104 (58.1) | 3.79 | 0.15 |
| Religiosity  Secular  Religious/very religious | 10 (11.5)  20 (9.2) | 19 (21.8)  74 (33.9) | 58 (66.7)  124 (56.9) | 4.32 | 0.11 |

**Supplemental material 3**: Differences in syndemic severity by gender and socioeconomic variables, with Cohen's *d*

| Variable | M(SD) | t | *p*=* | Cohen's *d* |
| --- | --- | --- | --- | --- |
| Gender^±^  Men  Women | 6.28 (2.81)  5.65 (2.56) | 2.05 | 0.04 | 2.67 |
| Marital status^¶^  Married  Not married | 5.80 (2.76)  6.08 (2.58) | -0.88 | 0.37 | - |
| Income  Below average/average  Above average | 5.95 (2.62)  5.35 (3.77) | 0.81 | 0.41 | - |
| Education  Not academic  Academic | 9.19 (2.85)  5.73 (2.55) | 1.47 | 0.14 | - |
| Religiosity  Secular  Religious/very religious | 6.32 (2.71)  5.77 (2.6) | 1.62 | 0.10 | - |

^±^*t-test*

^¶^ANOVA

*two-sided
